# Efficacies and Second-Year Effects of SPLAT GM™ and SPLAT GM™ Organic Formulations

**DOI:** 10.3390/insects6010001

**Published:** 2014-12-23

**Authors:** Ksenia S. Onufrieva, Andrea D. Hickman, Donna S. Leonard, Patrick C. Tobin

**Affiliations:** 1 Department of Entomology, Virginia Polytechnic Institute and State University, Blacksburg, VA 24061, USA; E-Mail: ahickma@vt.edu; 2 Forest Service, U.S. Department of Agriculture, Forest Health Protection, Asheville, NC 28802, USA; E-Mail: dleonard@fs.fed.us; 3 School of Environmental and Forest Sciences, University of Washington, Seattle, WA 98195, USA; E-Mail: pctobin@uw.edu

**Keywords:** gypsy moth, pheromone, disparlure, persistence, second-year effect

## Abstract

Mating disruption is the primary control tactic used against the gypsy moth, *Lymantria dispar* (L.) (Lepidoptera: Lymantriidae) under the gypsy moth Slow the Spread (STS) program. In this paper, we present the results of the multiyear study designed to evaluate a new liquid SPLAT GM™ (ISCA Tech, Riverside, CA, USA) Organic formulation, which is approved by the USDA to meet National Organic Program Standards for use in organic certified farms, for its ability to disrupt gypsy moth mating, and to evaluate the environmental persistence of SPLAT GM™ and SPLAT GM™ Organic formulations. Environmental persistence of the pheromone beyond the year of application is a significant concern since STS relies on trap catch data to evaluate treatment success. The study was conducted in 2007–2012 in forested areas in Virginia and Wisconsin, USA. We observed that SPLAT GM™ Organic reduced gypsy moth trap catch by ≥90% for 10 weeks in a similar manner as SPLAT GM™ and Hercon Disrupt^®^ II (Hercon Environmental, Emigsville, PA, USA). Although we observed persistent effects in all products one year after application, the persistence observed in SPLAT GM™ and SPLAT GM™ Organic was significantly lower than that of Hercon Disrupt^®^ II plastic laminated flakes.

## 1. Introduction

Gypsy moth was introduced into North America in 1869 and remains one of the most severe and economically important forest pests in the US [1]. Its larvae are generalist folivores capable of feeding on over 300 species of deciduous and coniferous host trees [2], including highly preferred host species within the genera *Betula*, *Crataegus*, *Larix*, *Populus*, *Quercus*, *Salix*, and *Tilia* [3]. In addition to forest and shade trees, gypsy moth also poses a threat to a number of fruit and nut crops such as apple, apricot, blueberry, filbert, pear, pistachio, and plum [4]. Since its introduction outside of Boston, Massachusetts, gypsy moth has continued to spread to the south and west, however >70% of the area thought to be susceptible to gypsy moth remains uninfested [5]. Gypsy moth is managed along a leading invasion front under the gypsy moth Slow the Spread (STS) program, which aims to reduce spread rates by targeting isolated low-density colonies most likely to contribute to range expansion [6,7,8]. Since 2000, the STS program has reduced gypsy moth spread from historical rates of ≈21 km·per year [9] to less than 4 km·per year [10], which is estimated to have prevented *L. dispar* infestation on more than 400,000 km^2^ between 2000 and 2010 [1].

The dominant control tactic used in the STS program is mating disruption, which is used on >200,000 ha per year representing, on average, >75% of all the treated hectares [7]. Compared to other pest control tactics available to the STS program, which include chemical and biological pesticides, mating disruption is less expensive, less environmentally toxic, and produces no known adverse non-target effects. Mating disruption is achieved by creating a background level of synthetic sex pheromone in the environment that prevents flying males from finding calling flightless females. Mating disruption works through competitive attraction, false trail following, camouflage of the plume produced by calling females by the airborne artificial pheromone, desensitization, habituation, sensory imbalance or a combination of those [11,12]. Recent studies have also suggested that in addition to inhibiting mate location, the application of pheromone can also delay mating, which significantly decreases fertility and fecundity due to oocyte resorption, lower oocyte production and diminished capacity to store sperm in older females, as well as decreased fertilization success in older males [13,14,15].

There are currently two gypsy moth synthetic pheromone formulations registered for use in gypsy moth management programs, including STS: Hercon Disrupt^®^ II (Hercon Environmental, Emigsville, PA, USA) plastic laminated flakes and SPLAT GM™ (ISCA Tech, Riverside, CA, USA) paraffin wax [16,17]. A previous study conducted to evaluate various dosages and pheromone formulations showed that Hercon Disrupt^®^ II plastic laminated flakes and SPLAT GM™ paraffin wax formulations applied at 15 or 37.5 g·AI/ha effectively disrupted mating for >10 weeks [16], which sufficiently includes the 6 week period over which adults are generally present [18] and provides a margin of safety.

Unlike other pest control programs that may require multiple applications of pheromone per season [19,20], the synthetic formulation of the gypsy moth pheromone disparlure (*cis*-7,8-Epoxy-2-methyloctadecane) has a strong persistent effect such that only one application per season is needed [21]. In fact, gypsy moth management programs have to consider this persistent effect in the evaluation of treatment success, which is based upon trap catch data in the year after application. For example, disparlure has been shown to reduce trap catch and mating success of females by up to 70% and 90%, respectively, even one year after application [17,22].

One explanation for this persistence could be that the disparlure molecule is relatively large (MW = 282.504 g/mol) with a 19C chain, which is longer than the chains of pheromones in the majority of insects managed using mating disruption [20]. Therefore, disparlure exhibits zero-order release for longer time [23] and has a higher partitioning coefficient compared to some of the other synthetic pheromones, which leads to creation of significant persistent or “ghost” effect [24]. This is also supported by reports on long-term absorption and re-emission of disparlure from human tissues [25,26]. Although this prolonged persistent effect can be viewed as an additional benefit of the applied treatment, in some cases it has been shown to interfere with the treatment evaluation, thus leading to an underestimation of pest population density [21]. This underestimation of the residual population could be particularly problematic when mating disruption is used in gypsy moth eradication programs. In eradication programs it is critical to detect populations while they are localized and their densities are low [27]. Failure to detect a new population or correctly estimate its density would likely lead to its spread, and increase cost and difficulty of eradication efforts [27,28].

Previous studies conducted to evaluate the persistent effects of synthetic pheromone applications suggested that liquid or biodegradable formulations may evaporate or biodegrade more quickly and therefore produce weaker second-year effect compared to plastic laminated flake formulation [22]. In this paper, we evaluated a new SPLAT GM™ Organic formulation that is approved by the USDA to meet National Organic Program Standards for use in and around organic certified farms. We compared the efficacy of the SPLAT GM™ Organic formulation with that of the two currently available formulations used in STS, SPLAT GM™ and Hercon Disrupt^®^ II plastic flakes; we also evaluated the persistent effects of SPLAT GM™ and SPLAT GM™ Organic formulations and compared them to persistent effect of Hercon Disrupt^®^ II. We conducted our field experiments in Wisconsin and Virginia, which represent northern and southern extremes of gypsy moth management programs in the US, to determine if product efficacy and persistence were influenced by different summer weather conditions.

## 2. Experimental Section

### 2.1. Study Sites

Field experiments were conducted in the Goshen Wildlife Management Area in Rockbridge county, VA, USA (38.0631° N, 79.3244° W to 38.0596° N, 79.3315° W), and in the Northern Highland American Legion State Forest in Oneida and Vilas Counties, WI, USA (46.1123° N, 89.4296° W to 45.9379° N, 89.6703° W) in 2010 and 2011. The primary objective was to evaluate the efficacy of SPLAT GM™ Organic and to compare its efficacy with that of SPLAT GM™ paraffin wax and Hercon Disrupt^®^ II plastic flakes.

### 2.2. Plot Layout and Pheromone Treatments

#### 2.2.1. Efficacy of SPLAT GM™ Organic

Studies were conducted in 2010 and 2011 to evaluate the efficacy of SPLAT GM™ Organic. For studies conducted in 2010, we selected eight plots in each state. Each plot was 500 × 500 m in size and separated from each other by at least 1 km of untreated area. Each plot was grouped into 2 blocks with 4 plots per block. In Wisconsin, one plot per block was an untreated control, while the other three were treated with either Disrupt^®^ II, SPLAT GM™ or SPLAT GM™ Organic at 15 g·AI/ha. In Virginia, one plot per block was an untreated control, one was treated with Disrupt^®^ II at 15 g·AI/ha, and the remaining plots were treated with SPLAT GM™ and SPLAT GM™ Organic. Due to an application error, SPLAT GM™ was applied at 11.4 g·AI/ha and SPLAT GM™ Organic was applied at 22.6 g·AI/ha. In both states, plots treated with Disrupt^®^ II and SPLAT GM™ served as positive controls. In Wisconsin, plots were monitored for 8 weeks, from 15 July to 27 August; in Virginia, plots were monitored for 18 weeks, from 17 June to 12 October.

In 2011, the experiments were only conducted in Virginia using six plots, each of which were 500 × 500 m in size, separated by 1 km of untreated area, and grouped into 2 blocks with 3 plots per block. In each block, one plot was an untreated control, one was treated with SPLAT GM™ and served as a positive control, and one was treated with SPLAT GM™ Organic. Due to an application error, both SPLAT GM™ and SPLAT GM™ Organic were applied at 9.9 g·AI/ha. Plots were monitored for 8 weeks, from 24 June to 12 August.

#### 2.2.2. Second-Year Effects of Aerially Applied SPLAT GM™ and SPLAT GM™ Organic

The treated and control plots used for evaluation of SPLAT GM™ in 2007–2008 [16] as well as control and treated plots used to evaluate SPLAT GM™ Organic in 2010–2011 were monitored in 2008–2009 and 2011–2012, respectively, to evaluate second-year effects of these formulations and to compare them with the second-year effect of Hercon Disrupt^®^ II. In each year, we used 2 male moth release points in each experimental plot that were located 150 m to the north and south of the plot center. Each release point was surrounded by 4 USDA milk carton pheromone-baited traps positioned 25 m away from the release point to prevent interference.

### 2.3. Pheromone Applications

The Disrupt^®^ II formulation (Hercon Environmental Corporation, Emigsville, PA, USA) consisted of plastic flakes composed of polyvinyl chloride (PVC) outer layers and an inner polymer layer containing 17.9% racemic disparlure ((*Z*)-7,8-epoxy-2-methyloctadecane). The flakes were mixed with diatomaceous earth (3% wt/wt) to reduce clogging and were aerially applied using a fixed-wing aircraft (Air Tractor) equipped with specialized application pods (Schweitzer Aircraft Corp., Elmira, NY, USA). Within the pods, the flakes were mixed with a multipolymer emulsion glue (Gelva 2333, Solutia Inc., Springfield, MA, USA) and dispensed through a spinner [17]. Disparlure release rate from applied flakes was not determined in this study. However, in previous studies where plastic flakes were applied under similar conditions, the flakes released 30%–50% of their disparlure content over the 6-week period of male moth flight [29,30].

SPLAT GM™ and SPLAT GM™ Organic are liquid disparlure formulations of emulsified paraffin wax (ISCA Technologies, Riverside, CA, USA) that are designed for both aerial and ground application. The formulation contains 13.0% racemic disparlure and is applied with conventional application systems pressurized either by positive displacement pumps, pressurized gas cylinders or a combination of both. SPLAT GM™ was applied using Beechcraft King Air aircraft. A Global Positioning Satellite (GPS) navigation system was used to guide all spray applications.

### 2.4. Treatment Evaluations

**Figure 1:**
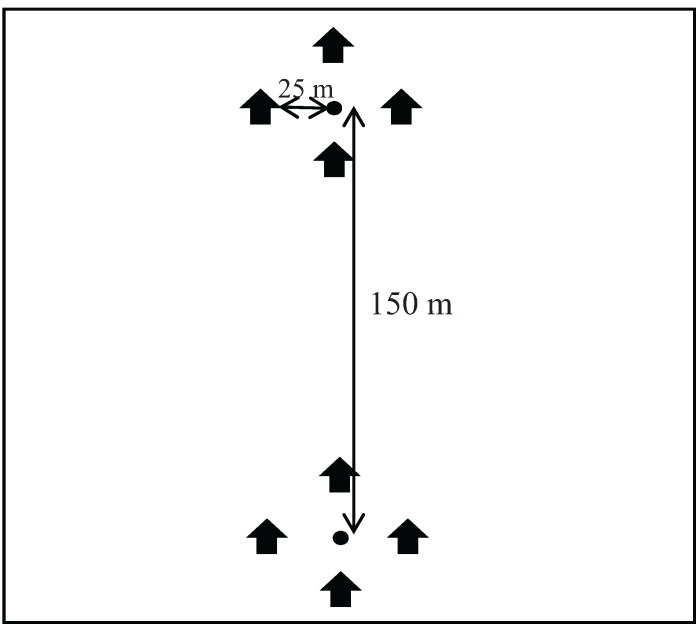
Experimental plot layout.

The efficacy of each treatment in disrupting mating was evaluated by release of laboratory-reared males. Male gypsy moths were obtained as pupae from the USDA Animal and Plant Health Inspection Service, Pest Survey Detection and Exclusion Laboratory, OTIS Air National Guard Base, MA, USA. Pupae were kept in laminated paper cups with plastic lids. A fluorescent dye solvent red 26 (Royce International, Paterson, NJ, USA) was added to the larval diet at the rearing facility, which was expressed in adults and could be used to differentiate between released and background male moths. Male moth recapture was determined using standard USDA milk-carton pheromone traps baited with 500 µg of (+)-disparlure in twine dispensers (Hercon Environmental Corporation, Emigsville, PA, USA) [31,32]. Each week, ≈150 adult males were released at each release point (Figure 1). Pheromone-baited traps were checked and emptied at the time of release. Male moths captured in pheromone-baited traps were removed and stored in the freezer. The moths were later examined under the microscope with a UV light for the presence of fluorescent dye. Only laboratory-reared, released males were used in statistical analyses to ensure equal male moth density among plots. The use of laboratory-reared, released males also allowed us to extend the time period during which the data could be collected since males could be released outside the feral males’ flight period.

●—male moth release point,  —pheromone-baited trap

### 2.5. Data Analysis

We used The General Linear Model ANOVA procedure with Tukey’s adjustment (JMP^®^ Pro 10, SAS Institute Inc., Cary, NC, USA, 2013.) to test for significance of differences in moth counts between groups of traps located in plots treated with various dosages and formulations of pheromone for each of the studies. Total moth counts per trap per week for each type of pheromone treatment were transformed using ln(N + 1), and we tested the main effects of week, dosage, and block as well as all possible interaction effects; the interaction of dosage and block was used as an error term.

## 3. Results

### 3.1. Efficacy of SPLAT GM™ Organic

**Figure 2:**
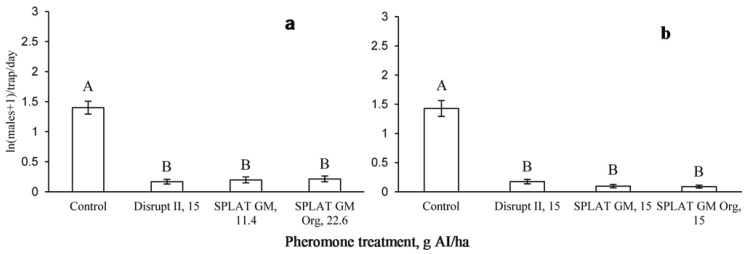
Male moth catches in pheromone-baited traps in (**a**) Virginia and (**b**) Wisconsin, 2010.

**Figure 3:**
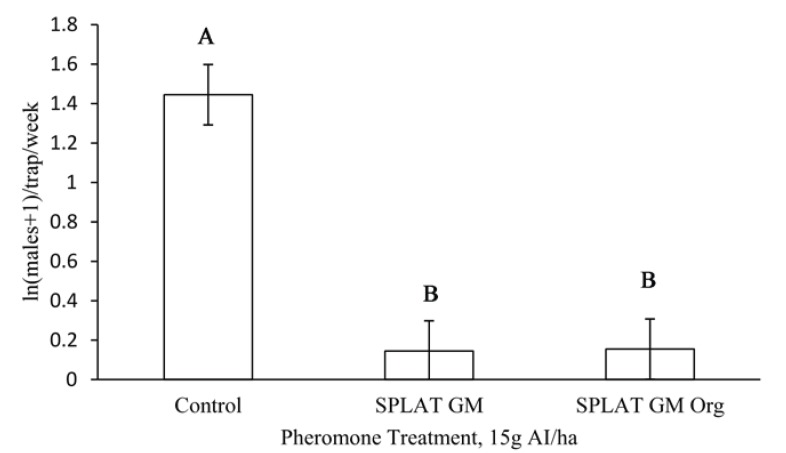
Male moth catches in pheromone-baited traps in Virginia, 2011.

In 2010, male moth catch in the pheromone-baited traps was significantly reduced by all treatments, relative to untreated control plots, in both Virginia (F = 64, df = 3, 84, *p* = 0.0013; Figure 2a) and Wisconsin (F = 29.7, df = 3, 124, *p* = 0.0099; Figure 2b). There was a significant effect of time (F = 2.9, df = 11, 72, *p* = 0.0038) on male moth trap catch when considering all 18 weeks over which this experiment was conducted in Virginia. However, both SPLAT GM™ and SPLAT GM™ Organic reduced male trap catch to the levels acceptable in STS for 10 weeks, which is considerably longer than the 6 week adult male flight period [18]. After 10 weeks, the efficacies of both formulations declined (Table 1). We observed the same pattern in 2011 in which male moth catch was significantly reduced by both treatments relative to untreated control plots (F = 55.4, df = 2, 46, *p* = 0.018; Figure 3).

**Table 1:** Decline in trap catch reduction (% of control) in experimental plots treated with various formulations and dosages of disparlure.

Weeks after Pheromone Application	Pheromone Treatment, g·AI/ha		
	SPLAT^®^ GM, 11.4	SPLAT^®^ GM Organic, 22.6	Disrupt^®^ II, 15
1–4	98%	98%	95.7%
5–8	~90%	~90%	94%
9–10	94.8%	92.8%	98%
11–14	85.8%	83.7%	
16–18	78.1%	79.7%	

### 3.2. Second-Year Effects of Aerially Applied SPLAT GM™ and SPLAT GM™ Organic

**Figure 4:**
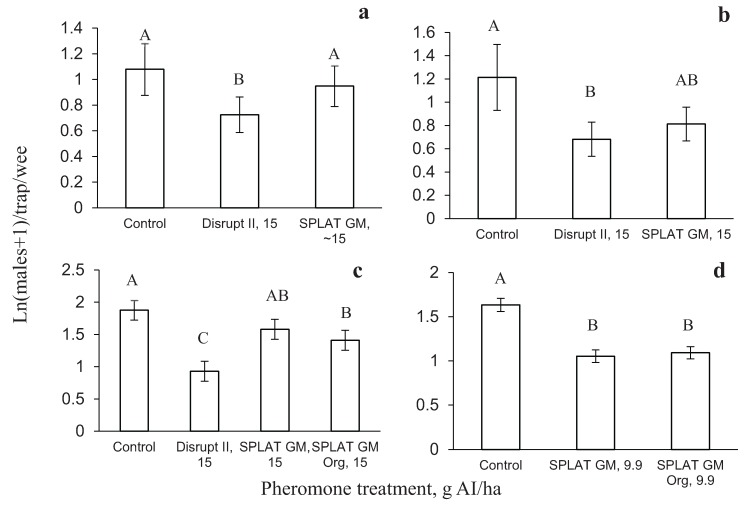
Male moth catches in pheromone-baited traps one year after pheromone application in (**a**) Virginia, 2008; (**b**) Virginia, 2009; (**c**) Wisconsin, 2011; and (**d**) Virginia, 2012.

In 2008, one year after the treatments were applied, male moth catch in pheromone-baited traps were reduced by 53% in plots treated with Hercon Disrupt^®^ II and by 29% in plots treated with SPLAT GM™. Mating success as measured by male moth catch in pheromone-baited traps was significantly reduced in plots treated with Hercon Disrupt^®^ II, but not in plots treated with SPLAT GM™ (F = 13.49, df = 2, 39, *p* = 0.0003; Figure 4a).

In 2009, one year after treatment application, male moth catch was reduced by 68% and 60% in plots treated with Hercon Disrupt^®^ II and SPLAT GM™, respectively. Similarly to 2008, mating success was significantly reduced in plots treated with Hercon Disrupt^®^ II, but not in plots treated with SPLAT GM™ (F = 4.68, df = 2, 32, *p* = 0.0003; Figure 4b).

In 2011, one year after treatment application, trap catch in treated plots in WI was still significantly suppressed in plots treated with Disrupt^®^ II and SPLAT GM™ compared to control plots, but not in plots treated with SLAT GM™ Organic (F = 13.2; df = 3, 52; *p* < 0.0001; Figure 4c). Trap catch in plots treated with Disrupt^®^ II, SPLAT GM™ and SPLAT GM™ Organic was suppressed by 70%, 44% and 30%, respectively, compared to untreated control plots. In 2012, trap catch was significantly reduced by both treatments applied in 2011 (F = 20.6, df = 2, 40, *p* < 0.0001, Figure 4d). Trap catch was reduced by 57% and 56% by SPLAT GM™ and SPLAT GM™ Organic, respectively.

## 4. Discussion

We evaluated the efficacy of a new SPLAT GM™ Organic flowable formulation and compared it with two products currently available for gypsy moth management programs, SPLAT GM™ flowable and Hercon Disrupt^®^ II plastic flakes. We also investigated the persistent effects of both SPLAT GM™ and SPLAT GM™ Organic formulations in the year after application. Lastly, by conducting our experiments in Wisconsin and Virginia, we determined if product efficacy and potential persistence were consistent between these two climatically-different regions.

The results of all studies indicated that SPLAT GM™ Organic formulation was as effective as SPLAT GM™ and Hercon Disrupt^®^ II when applied at similar dosages, irrespective of climate zone, and that it reduced mating success, as measured by male moth catch from pheromone-baited traps, by >90% compared to control plots. The results of the longevity experiment indicated that both SPLAT GM™ and SPLAT GM™ Organic reduced trap catches by ≥90% for 10 weeks, after which their efficacies started to decline.

To successfully disrupt mating in support of management programs, synthetic pheromones must be present in the air in sufficient quantities for the entire period of sexual activity of adults [33,34]. In the gypsy moth STS program, standard operating procedures require that pheromone applications reduce trap catch by at least 90% for a period of at least 8 weeks to cover the entire period of gypsy moth flight [17], which generally occurs up to 6 weeks [18]. The 8 week period of effectiveness also provides for a safety margin given the uncertainties associated with the logistics of treatment planning and application, and with the challenges in estimating the timing of gypsy moth adult flight given year-to-year variation in weather [17,18]. Therefore, SPLAT GM™ Organic satisfies the criteria for operational use in gypsy moth management programs, including STS, and its organic properties allow for its use in and around organic certified farms and other sensitive areas.

The results of the study conducted in Virginia (2008 and 2009) designed to evaluate second-year effects indicated that even though male moth trap catch in pheromone-baited traps in plots treated with SPLAT GM™ one year prior to evaluation ranged from 29% to 60% relative to control plots, they were not significantly different from trap catch in control plots. In 2012, trap catch in plots treated with SPLAT GM™ and SPLAT GM™ Organic one year prior to evaluation were reduced significantly, by 57% and 56%, respectively, compared to untreated control plots. In Wisconsin, SPLAT GM™ and SPLAT GM™ Organic reduced trap catch by 44% and 30%, respectively, and the differences between trap catch in control plots and plots treated with SPLAT GM™ Organic were not significant. In contrast, trap catch in plots treated with Hercon Disrupt^®^ II at 6 g·AI/acre one year prior to evaluation, were always significantly reduced compared to control plots. The trap catch reduction ranged from 46%–68% in Virginia and 70% in Wisconsin [21,22]. In a vast majority of cases, trap catch in plots treated with Hercon Disrupt^®^ II were significantly lower than in plots treated with SPLAT GM™ or SPLAT GM™ Organic.

Based on the lack of consistency in significant differences in trap catch between untreated control plots and plots treated with SPLAT GM™ and SPLAT GM™ Organic, we conclude that SPLAT GM™ and SPLAT GM™ Organic produce somewhat weaker second-year effects than Hercon Disrupt^®^ II. Both SPLAT GM™ formulations are emulsified paraffin wax dispensers that consist primarily of wax and water, and are applied as mixtures of dollops of different sizes. The speed of dollop degradation depends on dollop size as well as weather conditions, such as temperature and exposure to UV radiation [24]. This may explain significant variability in the second-year effects of SPLAT GM™ treatments.

The persistence of synthetic pheromones applied as part of insect pest management programs has been previously reported [21,22,35,36]. However, the exact mechanisms of pheromone persistence in the environment are not fully understood. Short-term persistence could result from environmental contamination in which bark, foliage, and leaf litter adsorb and re-emit pheromone over time, while long-term pheromone persistence has been linked to the controlled-release dispensers that remain on the ground and continue to release pheromone beyond the year of application [22,24,35,36,37]. For example, both SPLAT GM™ and SPLAT GM™ Organic are flowable emulsified paraffin wax formulations, and when applied in the field, the wax adheres to tree bark or foliage, releases pheromone for an extended period of time, and eventually erodes from bark and biodegrades in soil [38]. Previous studies conducted on disparlure persistence in the environment indicated that the long-term effect came exclusively from the dispensers left on the ground [22]; therefore, the results of this study suggests that in case of SPLAT paraffin wax formulations, the persistent effect is likely the result of release from the wax particles that have not completely biodegraded. However, the fact that wax degrades faster than plastic suggests that persistent effects of SPLAT GM™ formulations would be less than the persistence of plastic-based synthetic pheromone products.

## 5. Conclusions

a. SPLAT GM™ Organic reduced gypsy moth trap catch by ≥90% for 10 weeks in a similar manner as SPLAT GM™ and Hercon Disrupt^®^ II (Hercon Environmental, Emigsville, PA, USA).

b. The persistence observed in SPLAT GM™ and SPLAT GM™ Organic was significantly lower than that of Hercon Disrupt^®^ II plastic laminated flakes.
